# Internet Addiction and Depression in Chinese Adolescents: A Moderated Mediation Model

**DOI:** 10.3389/fpsyt.2019.00816

**Published:** 2019-11-13

**Authors:** Xinli Chi, Xiaofeng Liu, Tianyou Guo, Mingxia Wu, Xiaochen Chen

**Affiliations:** ^1^College of Psychology, Shenzhen University, Shenzhen, China; ^2^The Department of Psychology, Renmin University of China, Beijing, China; ^3^The Laboratory of the Department of Psychology, Renmin University of China, Beijing, China; ^4^Shenzhen Key Laboratory of Affective and Social Cognitive Science, Shenzhen University, Shenzhen, China; ^5^Southwest University Faculty of Psychology, Research Center of Mental Health Education in Southwest University, Chongqing, China

**Keywords:** internet addiction, depression, positive youth development, mindfulness, Chinese adolescents

## Abstract

Research has revealed that Internet addiction is a risk factor for adolescents’ development of depressive symptoms, although the underlying mechanisms are largely unknown. The present study examines the mediating role of positive youth development and the moderating role of mindfulness to determine the association between Internet addiction and depression. A sample of 522 Chinese adolescents completed measures related to Internet addiction, positive youth development, mindfulness, depression, and their background information, for which the results reveal that positive youth development mediates the relation between Internet addiction and depression. Moreover, the associations between both Internet addiction and depression as well as positive youth development and depression are moderated by mindfulness. These two effects were stronger for adolescents with low mindfulness than for those with high mindfulness. The present study contributes to a more thorough understanding of how and when Internet addiction increases the risk of depression in adolescents, suggesting that Internet addiction may affect adolescent depression through positive youth development and that mindfulness can alleviate the negative effect of Internet addiction or a low level of psychological resources on depression. The implications for research and practice are finally discussed.

## Introduction

The Internet has introduced significant convenience to our lives, although it has also brought about a serious mental health problem: Internet addiction, which is defined as an individual’s inability to control his/her use of the Internet (1, 2). Internet addiction may cause various psychological and behavioral problems in an individual’s life, including academic failure (3), sleeplessness (4), loneliness (5), and interpersonal relationship conflicts (6), among others. In recent years, depression has emerged as another prevalent and severe psychological problem in adolescents. Depression may negatively influence adolescents in various ways, including their achievement of poor interpersonal relationships, a low quality of life, academic failure, and even suicide (7–9). The relationships between Internet addiction and depression among adolescents are especially important because they are harmful (10), and evidence suggests that Internet addiction and depression are strongly correlated (11). For example, a study of 34 diverse high schools in Western Australia (12) found that Internet addiction (e.g., social networking sites addiction) may lead to depression, while a study of six Asian countries (13) determined that Internet addiction may positively predict depression in adolescents aged twelve to eighteen years.

Despite previous study findings that claim Internet addiction may be strongly related to depression, the underlying mediating mechanism (i.e., how Internet addiction influences depression) and moderating mechanism (i.e., when Internet addiction is related to depression, or the difference in the degree of Internet addiction related to depression among different groups) remain unclear, especially among Chinese adolescents. Regarding Internet addiction and depression in Chinese adolescents, reports have found that the prevalence of Internet addiction among adolescents aged eleven to eighteen years in China was between 2.4% and 18.2% (4, 14), while the prevalence of depression was about 30% among adolescents aged twelve to nineteen years (15). These serious problems are attracting considerable attention from society and mandating that research urgently explore the underlying mechanisms of Internet addiction and depression among Chinese adolescents. Therefore, the present study constructs a moderated mediation model to examine the mediating role of positive youth development and the moderating role of mindfulness in the relation between Internet addiction and depression in Chinese adolescents. The findings promote an understanding of how and when Internet addiction is associated with depression in adolescents so as to provide effective prevention and intervention strategies against Internet addiction and depression among this group.

### Positive Youth Development as a Mediator

The development assets theory suggests that the occurrence of problem behaviors may be attributed to a lack of positive psychological resources, such as positive youth development features (e.g., self-efficacy and resilience) (16). Psychological resources may be weakened by adversity, difficulties, and stress from environmental and personal perceptions (17–20). When these positive resources are absent, individuals no longer have the ability to adapt to their situations and are thus unable to rid themselves of the adverse effects of negative influence, which may further yield externalized and internalized problems (21, 22). Positive youth development, which integrates multiple psychological resources, refers to the individual’s potential in terms of talent, strength, interest, ambition, and so forth rather than one’s lack of abilities (23). Based on the positive youth development perspective, Catalano et al. (24) proposed a model comprising the following fifteen constructs: bonding, resilience, social competence, emotional competence, cognitive competence, behavioral competence, moral competence, self-determination, self-efficacy, spirituality, beliefs in the future, clear and positive identity, recognition for positive behavior, prosocial involvement, and prosocial norms. This model, which has been implemented by researchers in Western countries for youth development research, inspired Shek and Sun (25) to develop the Chinese Positive Youth Development Scale (CPYDS), which has been widely employed in the Chinese context (26, 27).

An increasing number of empirical studies demonstrate that positive youth development features may help prevent/reduce various externalized and internalized problems among adolescents (24, 28). Further, positive youth development constructs (e.g., positive identity and cognitive–behavioral competence) mediate the correlation between adversity (e.g., childhood maltreatment) and adolescent depression (28). Regarding Internet addiction and depression, researchers stress that Internet addiction can undermine adolescents’ offline activities by, for instance, influencing grade declines and poor parent–child and peer relationships, thus forcing them to experience stress (29). On the other hand, being tempted by the network and resisting the temptation to control themselves also force adolescents to experience stress. These stressors may result in their loss of psychological resources (e.g., self-efficacy) and increase their risk of developing mental health problems (e.g., depression) (30). Studies demonstrate that Internet addiction and depression are strongly associated with positive youth development; for example, Shek and Yu (31) found that positive youth development is negatively related to Internet addiction among adolescents aged ten to eighteen years in Hong Kong. Studies have also found that positive youth development negatively predicts depression (32, 33). Based on the development assets theory and previous research, we hypothesized that positive youth development mediates the relationship between Internet addiction and depression (Hypothesis 1).

### Mindfulness as a Moderator

Mindfulness refers to the state of being aware of the present reality or current experience in an accepting or non-judgmental way (34, 35). Some researchers believe mindfulness can be defined as a psychological trait that refers to an individual’s tendency to be mindful in one’s daily life (34, 36, 37); in other words, mindfulness may be simultaneously regarded as both a state and a trait. Previous studies have determined that mindfulness can be wielded as a protective factor during an individual’s positive development (38, 39). For example, Chen et al. (40) and Meiklejohn et al. (41) consistently find that mindfulness can promote adolescents’ mental health by, for instance, improving their resilience and emotional competence (e.g., emotional adjustment skills).

Although Internet addiction can negatively affect adolescents, this influence may vary according to age, gender, and personal traits (42, 43). We are interested in whether or not Internet addiction influences mental health differently among individuals with different levels of mindfulness. According to the re-perceiving model of mindfulness, mindfulness can help people re-perceive moment-by-moment experiences with greater objectivity and awareness, rid themselves of automatic behavioral and emotional patterns, and facilitate their adaptive responses to negative stimulation (44). Therefore, researchers suggest that mindfulness may play a risk-buffer role and alleviate the negative effects of risk factors on mental health (39, 45–47). Previous studies demonstrate that higher levels of mindfulness may simplify an individual’s development of the ability to re-perceive and subsequently reduce psychological distress as well as the effects of adversity or stress on one’s psychological health (48, 49). For example, mindfulness can weaken the impact of psychological distress on emotional eating behaviors (50) as well as alleviate the impact of bullying on mental resilience and depression (47).

These studies further determined a stronger relation between psychological vulnerability and mental health problems for individuals who possess lower mindfulness (45, 46, 47, 50, 51). Regarding the moderating effects of Internet addiction and mental health, research has determined that mindfulness may contribute toward alleviating the negative effects of Internet addiction (e.g., mobile phone addiction) on mental health (52). For example, mindfulness moderates the relation between mobile phone addiction and sleep disturbance, and this effect is weaker in individuals with higher levels of mindfulness (46, 53). Another study suggests that the effect of mobile phone addiction on depression is moderated by mindfulness, wherein the impact is stronger among adolescents with lower levels of mindfulness (51). Thus, in the present study, one may reasonably expect mindfulness to act as an important protective factor that moderates the link between Internet addiction and positive youth development (Hypothesis 2), the link between Internet addiction and depression (Hypothesis 3), and the link between positive youth development and depression (Hypothesis 4).

### The Present Study

**Figure 1:**
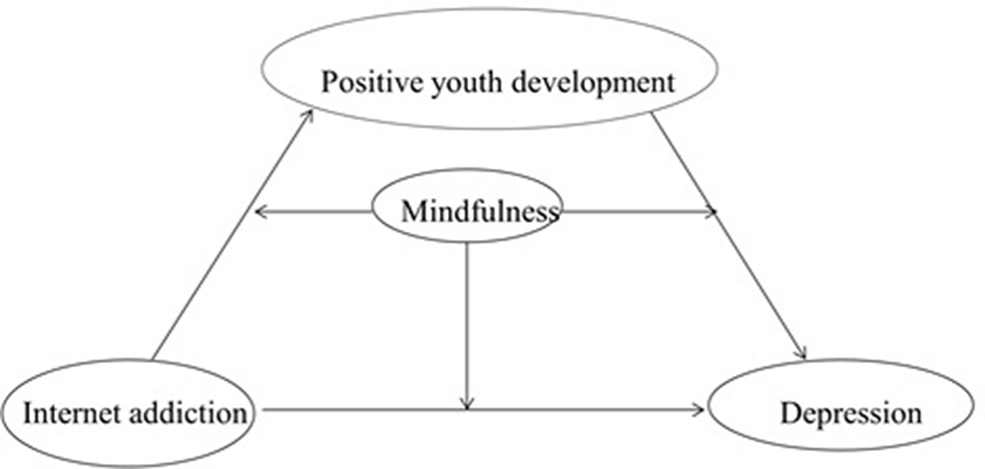
Conceptual model.

In summary, the present study examines the mechanisms underlying the association between Internet addiction and depression in adolescents as well as explores the mediating effect of positive youth development and the moderating effect of mindfulness. According to Hayes (54), regarding the combination of mediation and moderation models, if positive youth development mediates the association between Internet addiction and depression and mindfulness moderates the relation between Internet addiction and depression, the mediating effect of positive youth development is then moderated by mindfulness, and the moderated mediation model may be therein constructed. In this model, positive youth development acts as a mediator (i.e., how Internet addiction is associated with depression) while mindfulness acts as a moderator (i.e., when the relation between Internet addiction and depression is stronger) in the relationship between Internet addiction and depression. The proposed moderated mediation model is presented in **Figure 1** below.

## Method

### Participants and Procedure

A total of 532 adolescents from two public middle schools in China participated in our survey, of which 522 (98.12%) completed valid questionnaires. The participants comprised 298 male students and 221 female students. Three participants declined to report their gender, while three declined to report their age; the participants’ mean age was 12.33 (SD age = 0.56, range = 11–15). Two trained psychology graduate students administered the survey; one introduced the study’s purpose, while the other helped maintain order. During the roughly twenty-five survey, students were required to sit separately, to remain quiet, and to not engage in discussion. The study and data collection procedure received approval from the administration committees of the surveyed colleges and universities as well as the Human Research Ethics Committee of Shenzhen University.

### Measurements

#### Internet Addiction

Internet addiction was measured using Young and De Abreu’s (55) ten-item Internet addiction test. The participants rated these items by answering “yes” or “no” according to whether or not they had experienced the according Internet addiction in the past year. A person was classified as having an “Internet addiction” if he/she expressed four or more of the listed behaviors. The test achieved good reliability and validity in prior studies (33); in this study, the Cronbach’s α for Internet addiction was 0.82.

#### Depression

Depression was assessed using the Chinese version of the Center for Epidemiologic Studies Depression Scale (CES-D), which has been tested with Chinese adolescents and has reached good reliability and validity (56). The scale comprises twenty items, each of which was answered on a four-point scale (0 = never, 3 = always) wherein higher values indicate more severe depressive symptoms. The total depression score was divided by 16 points, 15 points, and less than 15 points for no depressive symptoms and 16 points or more for depressive symptoms. In this study, the Cronbach’s α for the CES-D was 0.87.

#### Positive Youth Development

Chinese Positive Youth Development Scale was compiled by Shek and Sun (25) in the Chinese culture context to examine adolescents’ fifteen positive psychological qualities (e.g., resilience, self-efficacy, emotional competence, cognitive competence) by scoring their responses on a scale ranging from 1–6 (1 = strongly disagree, 6 = strongly agree). The total score is averaged by items to form a scale score, with higher scores reflecting a higher level of positive psychological qualities. This scale is widely applied and has reached good reliability and validity in prior research (57). In this study, the Cronbach’s α for positive youth development was 0.97.

#### Mindfulness

Mindfulness was measured using the ten-item Child and Adolescent Mindfulness Measure (CAMM) (58), which has been implemented with Chinese adolescents and has reached good reliability and validity (59). This measure was scored on a scale ranging from 0–4 (0 = never true, 4 = always true). All items were scored in reverse, with higher scores indicating a higher tendency to be mindful in everyday life. In this study, the Cronbach’s α for the CAMM was 0.88.

### Statistical Analysis

In this study, to maximize statistical power, replacement values for missing data were first estimated using the expectation-maximization algorithm as implemented in SPSS 22.0. Descriptive statistics and a bivariate correlation analysis were secondly conducted. Third, the mediation model (model 4) and the moderated mediation model (model 59) were then tested by using the SPSS macro PROCESS. Why we choose SPSS macro PROCESS rather than SEM program is based on following considerations. Firstly, SPSS is still one of most popular tools used by researchers in psychology and psychiatry (many other fields as well), although new data analysis tools (e.g., Mplus) appear (60). Further, the SPSS macro PROCESS introduced by Hayes (54) has been widely utilized to test complex models of the observed variables, such as the moderated mediation model and the mediated moderation model (e.g., 46, 60, 61). One of advantages of SPSS macro PROCESS is that may offer the Johnson Neyman method of visualizing the interaction effect by generating a series of plots that can be later assembled into a diagram/graph. The diagram depicts the conditional effect of X (focal predictor) on Y (dependent variable), as a function of M (moderator variable). The moderating effects may clearly be probed using the regions of significance in accordance to the Johnson-Neyman technique (54). The bootstrapping method was applied to test for the effects’ significance so as to obtain robust standard errors for parameter estimation (54). Specifically, this method produced 95% bias-corrected confidence intervals for these effects from 1,000 resamples of the data; confidence intervals that do not contain zero indicate effects that are significant.

## Results

### Preliminary Analyses

The results of the descriptive statistics (mean and SD) and correlation analysis are presented in **Table 1**. In this study, the prevalence rates of Internet addiction and depression among adolescents were about 20.44% and 28.16%, respectively. Internet addiction was negatively correlated with mindfulness and positive youth development although positively correlated with depression. Positive youth development was negatively correlated with depression and positively correlated with mindfulness, while mindfulness was negatively correlated with depression.

**Table 1:** Descriptive statistics and inter-correlations between variables.

Variable	Not addicted/depressive	Addicted/depressive	M	SD	1	2	3	4
	*n* (%)	*n* (%)						
1. IA	327(79.56%)	84(20.44%)	1.54	2.18	–			
2. PYD	–	–	5.01	0.72	−0.33**	–		
3. Mindfulness	–	–	27.40	8.14	−0.39**	0.31**	–	
4. Depression	375(71.84%)	147(28.16%)	12.42	9.21	0.42**	−0.50**	−0.49**	–

N = 522, IA, internet addiction; PYD = positive youth development, **p < 0.01.

### Testing for the Mediation Model

To test the mediation model, we applied model 4 in model templates for the SPSS for SPSS (http://www.afhayes.com), as is suggested by Hayes (54). As can be perceived from **Table 2**, Internet addiction is positively associated with depression in the mediator’s absence (*β* = 0.46, *p* < 0.001), while Internet addiction is negatively associated with positive youth development (*β* = −0.33, *p* < 0.001). Moreover, when Internet addiction is controlled for, positive youth development is negatively correlated with depression (*β* = −0.40, *p* < 0.001), while when positive youth development is controlled for, the relationship between Internet addiction and depression is significantly positively (*β* = 0.28, *p* < 0.001). Finally, to test the mediation model, the bias-corrected percentile bootstrap method was conducted, and the present study generated 5,000 bootstrapping samples from the standardized data (*N* = 522) *via* random sampling. As can be observed in **Table 3**, the indirect effect of positive youth development was 0.13 (95% CI = [0.09, 0.18]); the empirical 95% confidence interval did not overlap with zero, which means the mediation effect was significantly. Further, the mediation effect accounted for 32.21% of the total effect of the relationship between Internet addiction and depression. Therefore, positive youth development mediates the association between Internet addiction and depression, and Hypothesis 1 is thereby supported.

**Table 2:** Mediation analysis.

Outcome variables	Independent variables	*B*	*SE*	*t*	*p*
Depression	Constant	0.0001	0.04	0.0001	1.00
	Internet addiction	0.46***	0.04	10.42	<0.001
PYD	Constant	0.0001	0.04	0.0001	1.00
	Internet addiction	−0.33***	0.04	−8.09	<0.001
Depression	Constant	0.0001	0.04	0.0001	1.00
	Internet addiction	0.28***	0.04	7.32	<0.001
	PYD	−0.40***	0.04	−10.37	<0.001

N = 522, bootstrap sample size = 5000; all data is standardized, the beta values are standardized coefficients, thus they can be compared to determine the relative strength of different variables in the model; ***p < 0.001.

**Table 3:** Bootstrapping indirect effect and 95% confidence interval (CI) for the mediation model.

Indirect path	Estimated effect	95% CI		Ratio to total effect on depression
		LL	UL	
Internet addiction→ PYD→ depression	0.13	0.09	0.18	32.21%

N = 522, bootstrap sample size = 5000, LL, low limit; CI, confidence interval; UL, upper limit; all data is standardized.

### Testing for the Moderated Mediation Model

To test the mediation model, we applied model 59 in model templates for the SPSS for SPSS (http://www.afhayes.com), as is suggested by Hayes (54). The results are displayed in **Table 4**, wherein the mediator variable model that predicts positive youth development indicates that Internet addiction is negatively associated with positive youth development (*β* = −0.27, *p* < 0.001), mindfulness is positively associated with positive youth development (*β* = 0.20, *p* < 0.001), and the interaction between Internet addiction and mindfulness is not significant (*β* = −0.02, *p* > 0.05); thus, hypothesis 2 is not supported. As can be seen from the dependent variable model that predicts depression, Internet addiction is positively correlated with depression (*β* = 0.12, *p* < 0.01), while the interaction between Internet addiction and mindfulness is negatively correlated with depression (*β* = −0.08, *p* < 0.05). Therefore, mindfulness moderates the relationship between Internet addiction and depression, and hypothesis 3 is thereby supported. In addition, positive youth development is negatively correlated with depression (*β* = −0.33, *p* < 0.001), while the interaction between positive youth development and mindfulness is negatively correlated with depression (*β* = 0.15, *p* < 0.001); namely, mindfulness moderates the relationship between positive youth development and depression, and hypothesis 4 is thereby supported.

**Table 4:** Conditional process analysis.

	*β*	*SE*	*t*	*p*
**Mediator variable model for predicting PYD**				
Constant	−0.01	0. 04	−0.18	0.86
Internet addiction	−0.27***	0.05	−5.57	<0.001
Mindfulness	0.20***	0.04	4.57	<0.001
Internet addiction x Mindfulness	−0.02	0.04	−0.55	0.58
**Dependent variable model for predicting depression**				
Constant	−0.07	0.04	−2.07	0.04
Internet addiction	0.12**	0.04	3.02	<0.01
Positive youth development	−0.33***	0.04	−9.13	<0.001
Mindfulness	−0.31***	0.04	−8.43	<0.001
Internet addiction x Mindfulness	−0.08*	0.03	−2.24	<0.05
Positive youth development x Mindfulness	0.15***	0.04	4.11	<0.001
	*B*	*SE*	*LLCI*	*ULCI*
Conditional direct effect analysis at values of the moderator				
M − 1SD	0.20***	0.04	0.11	0.28
M	0.12***	0.04	0.04	0.20
M + 1SD	0.05	0.06	−0.07	0.17
	*B*	*Boot SE*	*Boot LLCI*	*Boot ULCI*
Conditional indirect effect analysis at values of the moderator				
M − 1SD	0.12	0.03	0.06	0.18
M	0.09	0.03	0.04	0.14
M + 1SD	0.05	0.03	0.01	0.12

N = 522, bootstrap sample size = 5000, LL, low limit; CI, confidence interval; UL, upper limit; all data is standardized, the beta values are standardized coefficients, thus they can be compared to determine the relative strength of different variables in the model; *p < 0.05, **p < 0.01, ***p < 0.001.

**Figure 2:**
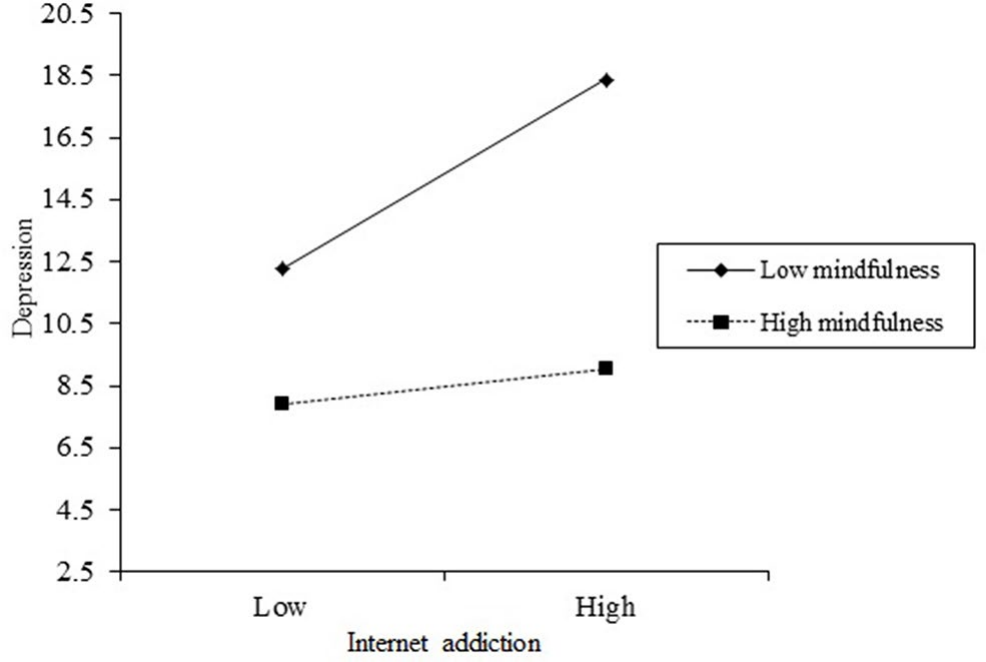
The moderating role of mindfulness in the relation between Internet addiction and depression. The moderating effect is graphed for two levels of mindfulness: (1) low mindfulness (1 SD below the mean) and (2) high mindfulness (1 SD above the mean).

**Figure 3:**
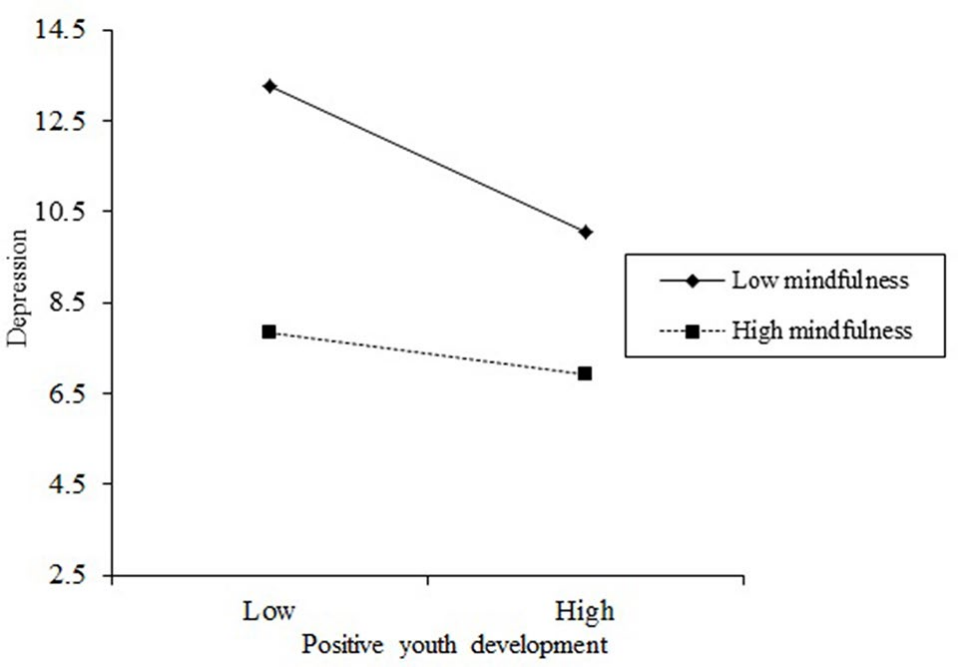
The moderating role of mindfulness in the relation between positive youth development and depression. The moderating effect is graphed for two levels of mindfulness: (1) low mindfulness (1 SD below the mean) and (2) high mindfulness (1 SD above the mean).

These results indicate that the relationships between both Internet addiction and depression as well as positive youth development and depression are moderated by mindfulness (see **Figures 2** and **3**). To more thoroughly understand the moderating effect of mindfulness, **Figure 2** describes the relationship between Internet addiction and depression at two levels of mindfulness (i.e., 1 SD below the mean and 1 SD above the mean). In addition, as can be observed from the conditional direct effect analysis in **Table 4**, the effect of Internet addiction on depression was observed when mindfulness was moderated to low (*β* = 0.20, *p* < 0.001), although not when mindfulness was high (*β* = 0.05, *p* > 0.05). Furthermore, to more thoroughly understand the moderating effect of mindfulness, **Figure 3** describes the relationship between positive youth development and depression at two levels of mindfulness (i.e., 1 SD below the mean and 1 SD above the mean). In addition, as can be seen from the conditional indirect effect analysis in **Table 4**, the effect of Internet addiction on depression was observed when mindfulness was moderated to low (*β* = 0.12, *p* < 0.001) as well as when mindfulness was high (*β* = 0.05, *p* < 0.05). In other words, positive youth development significantly predicted depression in adolescent group with low mindfulness, while among those with high mindfulness, positive youth development had a significant, but weaker prediction on depression.

## Discussion

In the present study, we constructed a moderated mediation model to analyze the mechanism underlying the association between Internet addiction and depression among Chinese adolescents. The results reveal that positive youth development plays the role of mediator and mindfulness plays the role of moderator in the relations between Internet addiction and depression and between positive youth development and depression; these two correlations are stronger for individuals with low mindfulness than for those with high mindfulness. These findings contribute to a more thorough understanding of how and when Internet addiction is associated with depression.

Specifically, we found that positive youth development mediates the relation between Internet addiction and depression. This result coincides with previous studies concerning the relationship between Internet addiction and positive youth development (11, 62) and the relationship between positive youth development and depression (32, 63–65). Researchers suggest that the displacement of offline social interaction *via* online social communication may lead to emotional disorders, such as depressive symptoms (66). For example, adolescents who engage in more online social activities may be more inclined to experience conflict with their parents and peers, social interaction withdrawal in real life, and a decline in their ability to regulate their emotions (67, 68). In other words, Internet addiction can deprive young people of forming real-world social relationships due to the excessive amount of time they spend online (13). These situations may reduce adolescents’ psychological strengths, such as their social, cognitive, and emotional competence, which may further intensify their withdrawal, avoidance, and negative feelings and consequently increase their risk of experiencing depressive symptoms (e.g., hopelessness and sadness) (69, 70).

In addition, we found that the direct link between Internet addiction and depression and the indirect effect of Internet addiction and depression through positive youth development are moderated by mindfulness. These two effects are stronger among adolescents with low mindfulness than those with high mindfulness, and these findings coincide with previous studies that attest to the risk buffer and protective function of mindfulness (45, 46, 48).

Firstly, the moderating effect of mindfulness in the relationship between Internet addiction and depression may be explained from the risk-buffer function of mindfulness; namely, the negative effects of Internet addiction may be buffered by mindfulness. Internet addiction is a risk factor not only for mental health (e.g., anxiety and sleep disturbances) (46, 51), but also for social maladjustment (e.g., poor academic performance and interpersonal problems) (71, 72). These stressors are likely to lead to adolescents’ psychological vulnerabilities, such as rumination as well as the refusal to accept and participate in real life, which may further lead to negative emotional experiences and increase their risk of developing depressive symptoms (73). Mindfulness refers to the state of being aware of the present reality or current experience in an accepting or non-judgmental way (34, 35), which may help adolescents rid themselves of rumination and enjoy their current lives and further reduce their likelihood of becoming depressed (74). By echoing the risk-buffer effect of mindfulness, the present study reports that Internet addiction negatively affects adolescents with low mindfulness more strongly than adolescents with high mindfulness. Thereby, adolescents with high levels of mindfulness may have a relatively greater capacity for ridding themselves of rumination and accepting the status quo, which in turn may reduce their risk of experiencing depression than those with low levels of mindfulness.

Second, the moderating effect of mindfulness in the relationship between positive youth development and depression may be explained from antagonistic interactions hypothesis of protective–protective model (75). Positive youth development (76, 77) and mindfulness (45, 47) are two protective factors that significantly contribute to mental health, for instance, by reducing an individual’s likelihood of experiencing depression; however, the effect of positive youth development on depression did not strengthen as mindfulness increased in our study, which is a result similar to those of previous studies that construct the moderated mediation model (45, 78). One explanation for this finding may be that mindfulness produces many positive effects on adolescent development, and adolescents with high mindfulness have fewer mental problems (e.g., depression). Thus, positive youth development does not express more positive effects for adolescents with high mindfulness. This finding may additionally indicate that positive youth development and mindfulness, as the two protective factors, may mutually compensate such as individual resource factors can buffer or weaken the adverse effects of risk factors. That is, highly positive youth development exhibits a stronger positive impact, while lowly positive youth development exhibits a stronger negative impact in adolescents with low mindfulness but not in adolescents with high mindfulness. Thus, adolescents with low mindfulness may rid themselves of negative impacts if they have highly positive youth development, and adolescents with lowly positive youth development may avoid negative impacts if they have high mindfulness.

Moreover, we did not find that mindfulness moderates the association between Internet addiction and positive youth development. This finding may imply that adolescents with either low or high mindfulness may be influenced by the negative effects of Internet addiction, which would more or less weaken their social and psychological competences. These findings are consistent with previous studies, thus confirming the adverse effects of Internet addiction on adolescents’ psychological resources (11, 45, 51, 62). Although adolescents’ psychosocial abilities are inevitably weakened by Internet addiction, the present study’s optimistic results assert that mindfulness may indeed alleviate or protect them from slipping into worse situations (e.g., depression). The findings may further imply that improving adolescents’ mindfulness may effectively alleviate the negative influence of Internet addiction on mental health.

## Limitations and Implications

The current study has several limitations. Firstly, the study utilized a cross-sectional research design that failed to prove causality between Internet addiction and depression. For this reason, future studies might wish to apply a longitudinal research design. Second, as the study was conducted in two secondary schools in Shenzhen, caution is urged in terms of the findings’ generalizability. Future studies may expand the survey’s scope by including a national scope. Finally, positive youth development includes four second-order constructs (i.e., prosocial attributes, positive identity, cognitive–behavioral competence, and general positive youth development qualities) (79), and we solely utilized the overall score of positive youth development as a mediator variable. Future research may attempt to respectively analyze the mediating effects of the four second-order constructs in the correlation between Internet addiction and depression.

Despite these limitations, the study has significant theoretical and practical implications. Firstly, the study tested the mediating role of positive youth development and the moderating role of mindfulness in the relationship between Internet addiction and depression, thus contributing toward a more thorough understanding of how and when Internet addiction is associated with depression in adolescents. The results suggest that parents and educators should help adolescents learn how to use the Internet responsibly. Educators may also wish to focus on helping adolescents increase their positive youth development traits (e.g., resilience, self-efficacy, and self-esteem) and mindfulness skills. These two aspects may promote adolescents’ adoption of positive coping strategies that enable them to deal with the negative impacts of Internet addiction. Previous studies reveal that positive youth development and adolescent mindfulness can be increased through training, such as Positive Adolescent Training through Holistic Social Programmes (80) and Mindfulness Based Cognitive Stress Reduction (81, 82). Therefore, according to previous studies, educators may wish to implement courses and activities that aim to improve positive youth development and mindfulness in an effort to reduce the negative impacts of Internet addiction on adolescents.

## Data Availability Statement

The datasets generated for this study are available on request to the corresponding author.

## Ethics Statement

Ethical review and approval was not required for the study on human participants in accordance with the local legislation and institutional requirements. Written informed consent to participate in this study was provided by the participants’ legal guardian/next of kin.

## Author Contributions

XCi and XL designed experiments, carried out experiments, conducted the statistical analysis and wrote the manuscript. TG, MW and XCe conducted data collection work and the double statistical analysis. XCe critically reviewed and revised the manuscript. All authors contributed to and have approved the final manuscript.

## Funding

This work was supported by fund for building word-class universities (disciplines) of Renmin University of China.

## Conflict of Interest

The authors declare that the research was conducted in the absence of any commercial or financial relationships that could be construed as a potential conflict of interest.
